# Evaluation of Polygenic Risk Scores for Prediction of Coronary Artery Disease in a Greek Case-Control Study

**DOI:** 10.3390/jpm14060565

**Published:** 2024-05-26

**Authors:** Maria Dimitriou, Panagiotis Moulos, Ioanna Panagiota Kalafati, Georgia Saranti, Loukianos S. Rallidis, George V. Dedoussis

**Affiliations:** 1 Department of Nutritional Science and Dietetics, School of Health Science, University of the Peloponnese, Antikalamos, 24100 Kalamata, Greece; 2 Institute for Fundamental Biomedical Research, Biomedical Sciences Research Center ‘Alexander Fleming’, 16672 Vari, Greece; 3 Department of Nutrition and Dietetics, School of Physical Education, Sport Science and Dietetics, University of Thessaly, 42132 Trikala, Greece; nkalafati@gmail.com; 4 Department of Nutrition and Dietetics, School of Health Science and Education, Harokopio University, 17671 Athens, Greece; dp4422213@hua.gr (G.S.); dedousi@hua.gr (G.V.D.); 5 Second Department of Cardiology, Medical School, National and Kapodistrian University of Athens, Attikon Hospital, 12462 Athens, Greece; lrallidis@gmail.com

**Keywords:** coronary artery disease, polygenic risk score, Greek, single-nucleotide polymorphisms, case-control, PRS, CAD, CVD, genetics

## Abstract

Coronary artery disease (CAD) stands as the most predominant type of cardiovascular disease (CVD). Polygenic risk scores (PRSs) have become essential tools for quantifying genetic susceptibility, and researchers endeavor to improve their predictive precision. The aim of the present work is to assess the performance and the relative contribution of PRSs developed for CVD or CAD within a Greek population. The sample under study comprised 924 Greek individuals (390 cases with CAD and 534 controls) from the THISEAS study. Nine PRSs drawn from the PGS catalog were replicated and tested for CAD risk prediction. PRSs computations were performed in the R language, and snpStats was used to process genotypic data. Descriptive characteristics of the study were analyzed using the statistical software IBM SPSS Statistics v21.0. The effectiveness of each PRS was assessed using the PRS R^2^ metric provided by PRSice2. Among nine PRSs, PGS000747 greatly increased the predictive value of primary CAD risk factors by 21.6% (*p*-value = 2.63 × 10^−25^). PGS000012 was associated with a modest increase in CAD risk by 2.2% (*p*-value = 9.58 × 10^−4^). The remarkable risk discrimination capability of PGS000747 stands out as the most noteworthy outcome of our study.

## 1. Introduction

Cardiovascular disease (CVD) mortality holds the foremost position globally, accounting for 32% of total worldwide deaths and 38% of premature deaths resulting from noncommunicable diseases (NCDs). Coronary artery disease (CAD) stands as the most predominant type of CVD with projections indicating a potential increase in CAD-related deaths to 23.6 million by 2030 [1,2].

Epidemiological studies have extensively documented the prevalence, incidence, and risk factors associated with CAD, providing critical insights into its burden on public health [3,4,5,6]. According to the updated statistical report from the American Heart Association (AHA), enhancements in low and moderate cardiovascular health (CH) levels underscore the potential to decrease or prevent premature CVD deaths. In order to improve CH outcome, AHA’s framework targets modifiable risk factors and promotes healthy lifestyle behaviors [7].

It is widely recognized that in addition to modifiable risk factors, CAD risk is also determined by the individual’s genetic composition. Furthermore, the bidirectional interaction between genetic predisposition and lifestyle and environmental factors can significantly affect CAD outcomes. Aligned with the overarching goal of preventing premature CVD mortality, genetic investigations have provided valuable insights into the polygenic nature of CVD with a particular emphasis on CAD. Genome-wide association studies (GWAS) represent notable contributions to understanding the genetic underpinnings of CAD [8,9,10,11]. Polygenic risk scores (PRSs) have emerged as valuable tools for assessing and quantifying genetic susceptibility to CAD, which is driven by advancements in statistical genetics and the discovery of CAD loci [12]. Therefore, PRS represents a quantitative assessment of an individual’s genetic susceptibility to complex traits or diseases derived from the cumulative effects of multiple genetic variants. Calculated by combining the effect sizes of individual variants proportionally weighted by their allelic contributions, PRS stands as a powerful tool for cardiovascular risk prediction [12,13,14].

The Polygenic Score (PGS) Catalog functions as a repository containing curated data on PRS models for various traits, including CAD “https://www.pgscatalog.org/ (accessed on 16 December 2023)” [15]. It includes comprehensive information on the traits or diseases investigated, the genetic variants incorporated into the scores, the populations or cohorts used for model development and validation, and the performance metrics assessing the predictive accuracy of the PRS [16]. The development of PRS models continues to progress as researchers validate them across diverse populations, ascertain their effectiveness across various genetic ancestries and endeavor to improve their predictive precision.

According to the existing literature, there is no information on specific PRS validated within the Greek population. The aim of the present work is to assess the performance and the relative contribution of PRSs specifically developed for CVD or CAD within a Greek population.

## 2. Materials and Methods

### 2.1. Study Population

The study population was drawn from the THISEAS (The Hellenic Study of Interactions between Single Nucleotide Polymorphisms and Eating in Atherosclerosis Susceptibility) database, constituting a case-control cohort. Information and details regarding the materials and methods of the study have been previously documented and published [17]. Cases consisted of patients with CAD, specifically with acute coronary syndrome or stable CAD, characterized by greater than or equal to 50% stenosis in at least one of the three major coronary arteries, which was confirmed through coronary angiography. Controls were individuals without CAD. To minimize the issue of misclassification, we obtained detailed information from medical histories via hospital or insurance records. Consequently, the control group consisted of individuals who had negative coronary angiography results, negative stress test results, or no clinical symptoms of cardiovascular disease. Exclusion criteria for both groups included the presence of renal or hepatic disease. Participants from the THISEAS database lacking genetic data or other essential parameters were excluded from current analyses.

Therefore, the sample under study for current analyses was restricted to 924 individuals, 390 diagnosed with initial CAD upon enrollment and 534 controls, depending mainly on the genetic data availability within the cohort. The study protocol received approval from the Ethics Committee of Harokopio University of Athens (approval protocol number and approval date: 10/9-6-2004 and 14-06-2004). Prior to providing their written consent, all participants were informed regarding the study.

### 2.2. Anthropometric Measurements

Body weight (BW) and height (Ht) were assessed in all participants who were dressed in light clothing and without footwear. BW was determined to the nearest 0.5 kg using a calibrated platform scale, while Ht was measured to the nearest 0.5 cm using a wall-mounted stadiometer. Body mass index (BMI) was calculated using Quetelet’s equation: BMI = BW (kg)/[Ht (m)]^2^.

### 2.3. Clinical Assessment

A physician conducted a clinical evaluation of the participants through a questionnaire administered during an interview. To mitigate recall bias, efforts were made to retrieve medical information from hospital or insurance records for both study groups whenever available. Hypercholesterolemia was defined as total cholesterol (TC) levels exceeding 200 mg/dl or the use of hypolipidemic medication. Individuals with diabetes were identified based on blood glucose levels greater than 125 mg/dl or if they were following a treatment regimen for diabetes. Blood pressure (BP) was measured in the right arm with the volunteer in a seated and rested position, using a mercury sphygmomanometer. Hypertension was defined as arterial BP levels equal or greater than 140/90 mmHg or if the individual was using antihypertensive medication.

### 2.4. Smoking Status Assessment

Participants were requested to report their smoking status as current, former, or never smokers. Current smokers were defined as individuals who smoked at least one cigarette per day, while non-smokers were those who had never smoked. Former smokers were defined as individuals who had ceased smoking for a minimum of six months. For current analyses, former smokers were categorized together with current smokers.

### 2.5. Genotyping

Whole blood samples were utilized for the extraction of genomic DNA (gDNA). For each participant, DNA isolation was conducted twice with each DNA sample stored in two aliquots with TE buffer at −20 °C. Genomic DNA samples were subsequently genotyped using the Illumina Omni-Express array 12V1, which is a customized genotyping array comprising 733,220 Single Nucleotide Polymorphisms (SNPs) (Human OmniExpress 12v1, Illumina, San Diego, CA, USA) [18]. Exclusion criteria for samples encompassed (i) sample call rates below 95%, (ii) samples with sex mismatches, (iii) individuals identified as ethnic outliers, (iv) samples exhibiting genome-wide heterozygosity levels deviating by more than ±3 standard deviations (SD), and (v) duplicate samples. SNP exclusion criteria included (i) deviation from the Hardy–Weinberg Equilibrium (HWE) with a *p*-value < 10^−4^ and (ii) call rates equal to or greater than 98%. After the quality control process, the rest of the dataset was expanded in terms of available markers by imputation. The latter was performed with IMPUTE2 [19] using the 1000 Genomes panel, phase 3 as a reference panel.

### 2.6. PRS

In total, nine PRSs were replicated, which were drawn from the PGS catalog (PGS catalogue accession IDs: PGS002437, PGS002486, PGS002535, PGS002584, PGS002633, PGS000012, PGS000116, PGS000337 and PGS000747) and tested for CAD risk prediction in the THISEAS database. PRS selection criteria were their relation with the CAD trait (as examined in the referenced publications in their PGS Catalog entries) and the referenced ancestries. Five PGSs (PGS002437, PGS002486, PGS002535, PGS002584, and PGS002633) originated from European ancestry populations in the UK Biobank, while four polygenic risk scores (PGS000012, PGS000116, PGS000337, and PGS000747) were derived from multi-ancestry populations. All PGSs have undergone prior evaluation in populations of European ancestry with the exception of PGS000337.

Prior to PRS calculations in the THISEAS population, the public PRSs from PGS Catalog were examined for compatibility with the replication data and sanitized accordingly. Specifically, markers that were present in the PGS Catalog PRSs but absent in our imputed data were replaced by proxies (LD > 0.8) “https://pubmed.ncbi.nlm.nih.gov/30024900/ accessed on 17 December 2023)”. Those markers for which no proxy candidates were found were dropped. Then, the public PRSs were examined with respect to our reference data regarding major and minor allele consistency and possible strand flips. Alleles were switched, when possible, which was accompanied by a reverse of the effects (weights) accompanying the retrieved PRSs. Then, the local (THISEAS data) PRS was computed by adding together the weighted values of each SNP assigned to every individual, multiplied by their genotype dosage, which was based on an additive model. The equation used to calculate PGS is:

$${\mathrm{PGS}}_{i}=\underset{i=1}{\sum }\hat{\beta_{j}}G_{\mathrm{ij}}$$

where PGS_i_, is the PGS for each individual, $\hat{\beta_{j}}$ is the effect size associated with each SNP, G_ij_ is the genotype value (0, 1, 2) for each SNP and for each individual in the study sample.

All calculations were performed in the R language. The R package snpStats was used to import and process genotypic data.

### 2.7. Statistical Analysis

Continuous variables were presented as means and SDs, while categorical variables were expressed as relative frequencies. Adjusted logistic regression analyses were conducted to assess the association between PRSs and CAD odds with odds ratios (ORs) and their corresponding 95% confidence intervals (CIs) calculated. The first model included sex, age, weight and type 2 diabetes mellitus (T2DM) as covariates. Model 1a (R^2^ without PRS, null model) included the aforementioned covariates, while Model 1b (R^2^ with PRS, full model) also included the PRS as a covariate. The models included 15 principal components (PCs) as covariates to capture additional population structures [20]. The number of PCs was automatically calculated using a proposed process based on the Tracy–Widom test “https://pubmed.ncbi.nlm.nih.gov/17194218/ (accessed on 18 January 2024)”.

$$\mathrm{Model}\;1a.\;\mathrm{Risk}=\beta_{1}\times \mathrm{sex}+\beta_{2}\times \mathrm{age}+\beta_{3}\times \mathrm{weight}+\beta_{4}\times T2D+\sum^{\;}\beta_{j}\times {\mathrm{PC}}_{j}$$

$$\mathrm{Model}\;1b.\;\mathrm{Risk}=\beta_{1}\times \mathrm{sex}+\beta_{2}\times \mathrm{age}+\beta_{3}\times \mathrm{weight}+\beta_{4}\times T2D+\sum^{\;}\beta_{j}\times {\mathrm{PC}}_{j}+\beta_{p}\times \mathrm{PRS}$$

The second model included seven covariates: namely, sex, age, weight, T2DM, systolic BP (SBP) levels and total cholesterol levels. These covariates are the main variables used in the calculation of the HellenicSCORE for CVD mortality [21]. Model 2a (null model) included the aforementioned covariates, whereas Model 2b (full model) also included PRS.

$$\begin{array}{ll} \mathrm{Model}2a & \\ \mathrm{Risk}=\beta_{1}\times \mathrm{sex} & +\beta_{2}\times \mathrm{age}+\beta_{3}\times \mathrm{weight}+\beta_{4}\times T2D+\beta_{5}\times \mathrm{smoking}+\beta_{6}\times \mathrm{SBP}+\beta_{7}\times \mathrm{total}\;\mathrm{cholesterol}\\ & +\sum^{\;}\beta_{j}\times {\mathrm{PC}}_{j} \end{array}$$

$$\begin{array}{ll} \mathrm{Model}2b & \\ \mathrm{Risk}=\beta_{1}\times \mathrm{sex} & +\beta_{2}\times \mathrm{age}+\beta_{3}\times \mathrm{weight}+\beta_{4}\times T2D+\beta_{5}\times \mathrm{smoking}+\beta_{6}\times \mathrm{SBP}+\beta_{7}\times \mathrm{total}\;\mathrm{cholesterol}\\ & +\sum^{\;}\beta_{j}\times {\mathrm{PC}}_{j}+\beta_{p}\times \mathrm{PRS} \end{array}$$

The variance explained in CAD risk for each PRSs was computed using the following equation:

$${\mathrm{PRS}\;\mathrm{adjusted}\;R}^{2}={\;R}^{2}\mathrm{model}\;\mathrm{with}\;\mathrm{PRS}-R^{2}\mathrm{model}\;\mathrm{without}\;\mathrm{PRS}$$

The descriptive characteristics of the study were analyzed using the statistical software IBM SPSS Statistics 21.0 (SPSS Inc., Frisco, TX, USA), and statistical significance was set at *p* < 0.05. To assess the effectiveness of each polygenic risk score, we utilized the PRS R^2^ metric provided by PRSice2 [22]. This metric quantifies the percentage of CAD risk elucidated by the PRS within the regression models. In this scenario, the statistical significance underwent adjustment via the Bonferroni correction method [23].

## 3. Results

The descriptive characteristics of the study are summarized in Table 1. Among 924 participants, 64% were men and 36% were women with a mean age of 58 years and a mean BMI of 28.3 kg/m^2^. The two study groups statistically differed regarding age (*p*-value < 0.01) and sex distribution (*p*-value < 0.01). The prevalence of T2DM and cigarette smoking was greater among cases than among controls (*p*-value < 0.001). The mean levels of systolic blood pressure and total cholesterol were observed to be higher among individuals in the control group compared to the case group (*p*-value < 0.001). Notably, the case group was receiving treatment for hypertension and dyslipidemia in higher rates than the control group. No differences were observed in terms of BMI among the two study groups.

Relevant information for each PRS evaluated in the present study is available in Supplementary Material, Table S1: Polygenic Risk Scores evaluated in the THISEAS study. Specifically, the Supplementary Material presents the PGS/PRS ID, PGS name, the number of genetic variants in each PGS, the associated trait, and the population sample sets used for evaluation [15]. Additional information such as the performance metrics, development methods and the studies describing the development and validation of these PGSs can be easily accessed online in the PGS Catalog database “https://www.pgscatalog.org/ (accessed on 16 December 2023)”.

The contribution of each PRS in total CAD risk is summarized in Table 2. Overall, PGS000747 and PGS000012 increased the predictive power of the model by R^2^ = 28% (*p*-value = 2.41 × 10^−78^) and R^2^ = 4.1% (*p*-value = 7.85 × 10^−13^), respectively. Smaller but significant increments in the odds of having CAD were reported for PGS000116 (R^2^ = 2.3%, *p*-value = 8.48 × 10^−8^) and PGS000337 (R^2^ = 3%, *p*-value = 9.55 × 10^−10^).

We further examined the predictive value of PGS PGS000747 and PGS000012 on CAD risk by adding more covariates as described in Model 2 in the Section 2. The results are depicted in Table 3. PGS000747 greatly increased the predictive value of primary CAD risk factors by 21.6% (*p*-value = 2.63 × 10^−25^). PGS000012 yielded a small increment of CAD risk by 2.2% (*p*-value = 9.58 × 10^−4^).

## 4. Discussion

PRSs, otherwise known as PGSs, are a predictive tool to estimate an individual’s genetic predisposition to various traits or diseases. Unlike monogenic traits or diseases influenced by a single gene mutation, polygenic traits such as CAD result from the cumulative effect of numerous genetic variants, each contributing a small effect to the disease. PRSs aggregate these effects to provide an overall risk score. Key components for PRS construction are (i) the genetic variants identified through GWAs studies, (ii) the effect sizes that depict the association strength between the SNP and the trait, (iii) the allele frequency of each SNP and (iv) LD adjustment to ensure that the score accurately depicts the aggregated effect of independent genetic variants [22,24].

The first step for PRS development involves data acquisition and compilation from GWAs studies conducted to demonstrate associations between SNPs and the disease of interest. The following steps involve the selection of the most predictive SNPs and PRS calculation by summing up the weighted effects of the selected SNPs. Finally, PRS validation and calibration in independent cohorts is crucial to estimate predictive accuracy and to ensure that predicted risks align to observed risks in the population [25].

The usefulness of PRSs is in their capacity to forecast genetic susceptibility. PRSs can stratify individuals into different risk groups based on their genetic predisposition. For instance, individuals within the highest decile of PRS distribution for CAD may have greater risk compared to those in the lowest decile [13,26]. Their predictive capability lies in providing a quantitative measure of genetic risk that can complement conventional risk factors. Although their utility in clinical settings is limited and demand responsible use, their prospective capacity will involve the identification of individuals at a young age who are at high risk and the implementation of tailored prevention strategies and treatment plans within clinical setting [12].

The predictive performance of PRSs can vary across populations due to differences in allele frequencies and LD patterns. Therefore, it is important to develop and validate PRSs in populations of diverse ancestries and ethnicities to ensure their applicability. Furthermore, it is essential to test combined models, integrating PRSs with traditional risk factors to improve the overall prediction model. For instance, combining PRS with clinical risk factors such as age, lifestyle and diabetes mellitus significantly enhances the prediction of CVD [13,27].

Given the above, in this analysis involving 924 individuals from a Greek case-control study, we aimed to replicate nine PRSs drawn from the PGS catalog to evaluate the impact of aggregated genetic data on CAD risk. Based on the findings of the present report, PGS000747 exhibited a notable contribution to distinguishing CAD risk, which was evident in both the baseline Model 1 (adjusted for sex, age, weight, and type 2 diabetes mellitus) and Model 2 (adjusted for sex, age, weight, type 2 diabetes mellitus, smoking, systolic blood pressure, and total cholesterol levels). Although PGS000012 also contributed to CAD risk discrimination, its effect was comparatively smaller.

Considering that the emergence of a PRS represents a significant advancement in assessing personalized risk beyond traditional risk factors, we conclude that the risk discrimination ability of PGS000747 stands out as the most important outcome of our study. PGS000747 consists of 375,822 variants and has undergone development and evaluation across diverse ancestral populations, including the European population. The reported trait under investigation for its development was CAD [24]. An interesting observation highlighted by Gola et al. (2020) emphasizes the necessity for PRS to be derived from its own specific training dataset or, at the very least, validated for applicability to the target population [28]. This underscores the significance of the present work, as it tested the performance of different PRSs tailored specifically for CAD within the Greek population, shedding light on which one demonstrates highest performance.

In clinical practice, it is recommended to assess an individual’s CVD risk and determine appropriate treatment initiation using risk assessment chart models. One such widely used model is the SCORE (Systematic COronary Risk Evaluation) tool, which was specifically designed for the European population [29,30]. The risk assessment tool specifically designed for the Greek population is known as the HellenicSCORE. This tool was developed based on national data and has undergone recent recalibration [21,31]. Similar to SCORE, the HellenicSCORE incorporates risk factors that influence CVD risk: namely, age, sex, smoking status, blood pressure and total cholesterol levels, as in Model 2 of our analysis. The addition of PGS000747 in our model significantly enhanced risk discrimination by 21.6%. Should this finding be replicated in subsequent cohorts, the development of an updated risk equation that incorporates genetic information would empower healthcare professionals to effectively identify individuals at greater risk of CVD and implement tailored preventive measures accordingly. Nonetheless, the Polygenic Risk Score Task Force of the International Common Disease Alliance (2021) has documented the potential advantages of integrating PRS into clinical practice. These scores offer numerous benefits, such as improved patient adherence and efficiency, better primary disease prevention—especially for individuals at greater risk or at younger ages—enhanced diagnostic accuracy, and increased precision in medication prescription [32].

A limitation of the current study is that the sample population primarily comprises volunteers recruited from a specific region (Attica). Although the sample size is not particularly small, its lack of representativeness for the Greek population is a limitation. Therefore, further research is needed, involving larger sample sizes and sub-populations within the country, to validate the findings of the current study. The findings of the present report add to the body of evidence regarding the validation of PRSs for applicability to the target population, thereby supporting progress in personalized medicine and precision healthcare initiatives.

The field of PRS research is rapidly evolving with promising directions. Integrating PRSs with other omics data (e.g., epigenomics, transcriptomics) could improve prediction accuracy and provide insights into the biological mechanisms of the underlying diseases. Advanced statistical and machine learning methods are explored to optimize SNP selection and weighting, potentially enhancing the predictive power of PRSs [33]. Research efforts to incorporate PRSs as useful tools in clinical practice will include the development of guidelines for the use of PRSs in risk assessment or/and the integration of PRS information in electronic health records.

## 5. Conclusions

Numerous studies have demonstrated the utility of PRS in identifying individuals at heightened risk for CVD, enabling early intervention and preventive measures. Notable research highlights the significance of PRS in refining risk stratification for CAD [12,13]. However, the clinical implementation of PRS necessitates the careful consideration of ethical, social, and practical implications [34]. As the field continues to evolve, integrating PRS into routine clinical practice holds promise for enhancing precision medicine strategies in managing cardiovascular health.

## Figures and Tables

**Table 1:** Descriptive characteristics of the study sample.

	THISEAS (n= 924)		Case Group (n = 390)		Control Group (n = 534)		*p*-Value
	Mean or Frequency	± SD *	Mean or Frequency	± SD *	Mean or Frequency	± SD *	<0.001
Age at assessment (years)	57.7 ± 12.4	±12.4	61.2	±10.3	54.6	±13.0	<0.001
Male sex (%)	63.9	-	82.8	-	50.0	-	<0.01
Body Mass Index (kg/m^2^)	28.3 ± 4.4	±4.4	27.85	±4.0	28.6	±4.7	0.344
Systolic blood pressure (mmHg)	132.6± 19.7	±19.7	131.5	±21.3	133.3	±18.6	<0.001
Prevalence of diabetes mellitus (%)	23.5	-	35.9	-	14.3	-	<0.001
Total cholesterol (mmol/l)	200.7 ± 44.6	±44.6	183.3	±47.1	212.0	±99.0	<0.001
Smokers (%)	65.0	-	79.0	-	52.9	-	<0.001

* SD = standard deviation; values are mean ± SD or n (%).

**Table 2:** PRSs’ contribution in overall CAD risk.

ID	R^2^	Adjusted PRS R^2^	PRS OR	*p*-Value
PGS002437	22.2%	<0.1%	1.016	0.706
PGS002486	22.2%	<0.1%	1.004	0.934
PGS002535	22.7%	0.5%	1.442	0.009
PGS002584	22.5%	0.3%	1.483	0.040
PGS002633	22.9%	0.7%	4.084	0.002
PGS000012	26.3%	4.1%	1.221	7.85 × 10^−13^
PGS000116	24.5%	2.3%	0.063	8.48 × 10^−8^
PGS000337	25.2%	3%	1.095	9.55 × 10^−10^
PGS000747	50.2%	28%	1.096	2.41 × 10^−78^

Adjusted PRS R^2^ = R^2^_model 1b_ − R^2^_model 1a_. Model 1a = adjusted for sex, age, weight and type 2 diabetes mellitus. Model 1b = Model 1 + PRS.

**Table 3:** PRSs’ contribution in overall CAD risk after adjusting for main variables of the HellenicSCORE.

ID	R^2^	Adjusted PRS R^2^	PRS OR	*p*-Value
PGS000012	41.4%	2.2%	1.153	9.58 × 10^−4^
PGS000747	60.8%	21.6%	1.095	2.63 × 10^−25^

Adjusted PRS R^2^ = R^2^_model 2b_ − R^2^_model 2a_. Model 2a = adjusted for sex, age, weight, type 2 diabetes mellitus, smoking, systolic blood pressure, total cholesterol. Model 2b = Model 2 + PRS.

## Data Availability

Summary statistics and data of this study are accessible upon request from the corresponding author. Participant data cannot be publicly available due to privacy concerns and ethical restrictions.

## Supplementary Materials

The following supporting information can be downloaded at: https://www.mdpi.com/article/10.3390/jpm14060565/s1, Table S1: Polygenic Risk Scores evaluated in the THISEAS study.
