# Supplementary material for: Evaluation of Polygenic Risk Scores for Prediction of Coronary Artery Disease in a Greek Case-Control Study
Source: J Pers Med. 2024 May 26;14(6):565. doi: 10.3390/jpm14060565 (PMC11204902; doi:10.3390/jpm14060565)
Supplement: Supplementary file 1 [file jpm-14-00565-s001.zip › jpm-3012664-supplementary.pdf]

Supplementary Table S1: Polygenic Risk Scores evaluated in the THISEAS study.

Authors	[*]PGS ID	PGS Name	Reported Trait	Number of SNPs	Derivation	PGS Evaluation (Sample Sets)
Weissbrod et al. (2022)	PGS002437	disease_CARDIOVASCULAR.P+T.0.001	CVD	20,431	[**]UKB European	African, East Asian, European, South Asian
Weissbrod et al. (2022)	PGS002486	disease_CARDIOVASCULAR.P+T.0.01	CVD	110,640	UKB European	African, East Asian, European, South Asian
Weissbrod et al. (2022)	PGS002535	disease_CARDIOVASCULAR.P+T.1e-06	CVD	1,218	UKB European	African, East Asian, European, South Asian
	PGS002584	disease_CARDIOVASCULAR.P+T.5e-08	CVD	637	UKB European	African, East Asian, European, South Asian
Weissbrod et al. (2022)	PGS002633	disease_CARDIOVASCULAR.PolyFun-pred	CVD	381,036	UKB European	African, East Asian, European, South Asian
Abraham et al. (2016)	PGS000012	GRS49K	CAD	49,310	Multi-ancestry	European, Multi-ancestry (including European)
Elliott et al. (2020)	PGS000116	CAD_EJ2020	CAD	40,079	UKB, Multi-ancestry	European, Multi-ancestry including European)
Koyama et al. (2020)	PGS000337	MetaPRS_CAD	CAD	75,028	Multi-ancestry	East Asian, Greater Middle Eastern
Gola et al. (2020)	PGS000747	PRS_EB	CAD	375,822	Multi-ancestry	European

[*]PGS= Polygenic Risk Score; [**]UKB= UK Biobank

Information regarding PGSs are drawn from the PGS catalogue online: https://www.pgscatalog.org/
